# Royal College of Surgeons

**Published:** 1846-05

**Authors:** 


*Successor to Breschet.*—The concours for the Chair of Anatomy in the Faculty of Medicine, rendered vacant by the death of M. Breschet, has terminated in the election of M. Denonvilliers by a majority of nine out of twelve votes. M. Chassaignac and Gosselin are reported to have highly distinguished themselves in this concours.—*Ibid.*

